# Anticancer Effects of Plasma-Activated Medium Produced by a Microwave-Excited Atmospheric Pressure Argon Plasma Jet

**DOI:** 10.1155/2020/4205640

**Published:** 2020-07-30

**Authors:** Ara Jo, Hea Min Joh, Tae Hun Chung, Jin Woong Chung

**Affiliations:** ^1^Department of Biological Sciences, Dong-A University, Busan 49315, Republic of Korea; ^2^Department of Materials Physics, Dong-A University, Busan 49315, Republic of Korea

## Abstract

Cold atmospheric plasma (CAP) has been reported to have strong anticancer effects *in vitro* and *in vivo*. CAP has been known to induce apoptosis in most cancer cells by treatment to cells using direct and indirect treatment methods. There are many reports of apoptosis pathways induced by CAP, but for indirect treatment, there is still a lack of fundamental research on how CAP can cause apoptosis in cancer cells. In this study, we applied an indirect treatment method to determine how CAP can induce cancer cell death. First, plasma-activated medium (PAM) was produced by a 2.45 GHz microwave-excited atmospheric pressure plasma jet (ME-APPJ). Next, the amounts of various reactive species in the PAM were estimated using colorimetric methods. The concentration of NO_2_^–^ and H_2_O_2_ in PAM cultured with cancer cells was measured, and intracellular reactive oxidative stress (ROS) changes were observed using flow cytometry. When PAM was incubated with A549 lung cancer cells, there was little change in NO_2_^–^ concentration, but the concentration of H_2_O_2_ gradually decreased after 30 min. While the intracellular ROS of A549 cells was rapidly increased at 2 hours, there was no significant change in that of PAM-treated normal cells. Furthermore, PAM had a significant cytotoxic effect on A549 cells but had little effect on normal cell viability. In addition, using flow cytometry, we confirmed that apoptosis of A549 cells occurred following flow cytometry and western blot analysis. These results suggest that among various reactive species produced by PAM, hydrogen peroxide plays a key role in inducing cancer cell apoptosis.

## 1. Introduction

Anticancer therapy using cold atmospheric plasma (CAP) is a rapidly developing field through collaboration with physics, biology, chemistry, and medicine [1–6]. CAP is an ionized gas near room temperature, which consists of various molecules, radicals, ions, electrons, excited species, electric field, and UV radiation. Previous studies have reported that CAP exhibited antimicrobial activity in bacteria [7–10] and induced angiogenesis by regulating wound healing molecular expression in skin cells [11–13]. In particular, CAP contains highly reactive oxygen and nitrogen species (RONS), and it can induce cell death by causing oxidative damage to cancer cells. CAP has been reported to disrupt oxidative balance [14, 15], induce DNA damage [16–19], and depolarize mitochondrial membrane potential [20].

In general, there are two ways to treat CAP to cells [21, 22]. First, direct treatment can induce strong cell death by reaching the plasma species to the cells. However, only very local treatments are possible, and toxic substances such as ozone can enter the normal cell directly. Second, indirect treatment is a method of inducing RONS in a solution by treating plasma to a medium or buffer. Although it causes less cell death than direct treatment, it has minimal effects on normal cells, is easy to process and store plasma-generated RONS, and can be treated on large areas. Thus, plasma-activated medium (PAM) generated by indirect treatment can be a good candidate for biomedical applications such as cancer therapy [23].

Our group has previously reported that the DNA damage ratio and cell death were increased by treating cancer cells directly with oxygen-containing CAP [24]. Additionally, cell death by ME-APPJ (788 MHz) utilizing a coaxial transmission line resonator (CTLR) was also confirmed [25]. As such, many studies on the mechanism by which CAP induces cancer cell death have been reported [26–33], but for the indirect treatment utilizing PAM, there is still a lack of research on physical changes in the stage before death occurs.

In the present study, we used atmospheric pressure plasma jets (APPJs). The use of APPJs in cancer cell treatment has received considerable attention because of convenient delivery of plasma species into the biotargets [34–36]. Among them, microwave-excited APPJs (ME-APPJs) can have high density of electron and reactive species, low breakdown power, low heavy-particle temperature, and lower discharge voltages [37–41]. It is expected that ME-APPJs are more efficient in generating RONS than their counterparts in low-frequency APPJs. Furthermore, the commercially available jet employed in this study could allow one to reproduce the experiments thereby standardizing plasma cancer therapy.

In order to investigate the anticancer effect by PAM, we used human lung cancer cells and human newborn foreskin fibroblasts to determine how PAM induced cancer cell death. As the working conditions of ME-APPJs, two flow rates (1.3 SLM and 1.9 SLM), three powers (5, 7, and 8 W), and three irradiation times (60 sec, 120 sec, and 180 sec) were employed. When plasma was applied to media, plasma properties were analyzed using the optical emission spectrum of the ME-APPJ. And then, the expression levels of NO_2_^–^ and H_2_O_2_ in PAM-treated cells were measured using colorimetric methods, and intracellular reactive oxidative stress (ROS) was also measured using an oxidative stress assay. Finally, cell death induced by PAM was evaluated by a flow cytometer analyzer and western blot analysis.

## 2. Materials and Methods

### 2.1. Cell Culture

For in vitro studies, human non-small-cell lung cancer A549 cells and human fibroblast Nuff cells were used. The cells were grown in DMEM medium supplemented with 10% fetal bovine serum and 1% penicillin/streptomycin (Capricorn Scientific, Ebsdorfergrund, Germany). The cells were maintained in the monolayer at 37°C in a humidified atmosphere with 5% CO_2_.

### 2.2. Plasma Sources and Preparation of PAM

In producing PAM, a 2.45 GHz commercial microwave-excited APPJ (PM-10, Heuermann HF-Technik GmbH) was used to treat the cell culture medium. The operating conditions of the ME-APPJ included various argon gas flow rates (1.3 and 1.9 SLM) and input powers (5–8 W). The optical emission spectrum and the gas temperature of ME-APPJ were measured using an optical spectrometer (USB-2000+XR-ES Ocean Optics) (for Stark broadening measurement, monochromator (SPEX 1702) with a photomultiplier tube (PMT) (Hamamatsu R928) was used) and a fiber optic temperature sensor (Luxtron M601-DM&STF), respectively. The cell culture medium was prepared to 3 mL in 60 mm Petri dishes and treated by plasma for 30 sec–180 sec. The distance between the open end of the quartz tube and the surface of the cell culture medium was fixed at 5 mm during plasma treatment. And then, the media on top of cells grown in plates were replaced by PAM.

### 2.3. Griess Assay

The Griess assay can measure the nitrite concentration and provide a good measure of the concentration of these nitrogen oxides. Nitrite concentration in PAM and PAM including A549 cells was determined with a Griess Reagent Kit (Molecular Probes) according to the manufacturer's directions. The absorbance at 548 nm was measured using a VersaMax™ Microplate Reader with SoftMax® Pro Software (Molecular Devices). The nitrite concentration was quantified utilizing a calibration curve prepared by using the standard sodium nitrite solutions (Molecular Probes, USA).

### 2.4. Measurement of H_2_O_2_ Production

To measure H_2_O_2_ quantification, we purchased the Amplex™ Red Hydrogen Peroxide/Peroxidase Assay Kit (Invitrogen, Carlsbad, USA). The experiment was conducted according to the manufacturer's protocol. On the day before PAM treatment, A549 cells were seeded at 7,000 in 96-well plates. The next day, PAM was treated with A549 cells at the desired time. At the end of treatment, 50 *μ*L of PAM was incubated with 100 *μ*M Amplex red reagent (10-acetyl-3,7-dihydroxyphenoxazine) and 0.2 U/mL HRP (horseradish peroxidase) in 1x reaction buffer (Krebs-Ringer phosphate buffer) for 30 min in the dark. Fluorescence was measured on FLUOstar OPTIMA (BMG LabTech, Ortenberg, Germany) with 544 nm excitation and 590 nm emission wavelengths.

### 2.5. Cell Viability Assay

In order to confirm the cytotoxicity of PAM in A549 cells, we used 3-(4,5-dimethylthiazol-2-yl)-2,5-diphenyltetrazolium bromide (MTT; Duchefa Biochemie, Haarlem, Netherlands). The A549 cells were seeded in 96-well plates at a density of 7,000 cells/well. After the cells are attached, PAM was treated at the desired time. After incubation, PAM was removed and 100 *μ*L MTT solution was added to each well, and the cells were further incubated for 3 hours. MTT produces a water-insoluble violet formazan by mitochondrial dehydrogenases of living cells. The violet crystals are solubilized with DMSO, and the absorbance was measured at 550 nm using a microplate reader. The relative cell viability (%) was calculated as (O.D.of PAM‐treated cells/O.D.of nontreated cells) × 100.

### 2.6. Intracellular ROS Detection

The percentage of intracellular ROS was measured using a Muse Oxidative Stress Assay Kit (Merck Millipore, Billerica, Mam USA), following the manufacturer's protocol. Briefly, A549 cells were incubated in 12-well plates (50,000 cells/well) with PAM for 1, 2, 3, or 24 hours at 37°C. At the end of treatment, the cells were washed with PBS buffer and collected in 1.5 mL microtube. Subsequently, the cells were incubated with the Muse Oxidative Stress working solution in the dark at 37°C for 30 min and quantified using the Muse Cell Analyzer.

### 2.7. Annexin V and Dead Staining

The experiment and procedures were described in detail [42]. Briefly, to detect apoptosis induced by PAM on A549 cells, we used the Muse Annexin V and Dead Cell Assay Kit. A549 cells were seeded in 12-well plates (50,000 cells/well). The next day, the cells were treated with PAM of various conditions and 100 *μ*M H_2_O_2_ (positive control) for 24 hours. The cells were harvested and incubated with 100 *μ*L Annexin V and Dead staining solution for 20 min at room temperature. Then, cells were analyzed using a Muse Cell Analyzer and Muse analysis software.

### 2.8. Cell Cycle Analysis

A549 cells were incubated in 12-well cell culture plates (50,000 cells/well) and allowed to attach for 24 hours. The cells were treated with PAM exposed to plasma for 0, 60, 120, or 180 sec, and 100 *μ*M H_2_O_2_ was treated for positive control. After the treatment was finished, all treated cells were collected and incubated with 200 *μ*L 70% EtOH for 3 hours at -20°C. After incubation, 70% EtOH was removed and a Muse Cell Cycle reagent was added to each microtube, and the cells were incubated at room temperature for 30 min. The ratio of cells in G0/G1, S, and G2/M phases was measured using the Muse Cell Analyzer.

### 2.9. Caspase-3/7 Analysis

For evaluating the caspase-3/7 level, A549 cells were seeded on 12-well plates and incubated for attachment. After removing the culture medium, PAM of different irradiation times were added followed by further incubation of 24 hours. Subsequently, cells were treated with Muse caspase-3/7 working solution for 30 min at 37°C in darkness. Samples were mixed using a vortex mixer and loaded onto the Muse Cell Analyzer, where the test of the degree of caspase-3/7 was performed following the user's guide protocol.

### 2.10. Western Blot Analysis

Cells were seeded and treated with PAM for 0.5 hours. At the end of treatment, cells were harvested and lysed in RIPA buffer. To measure the extract concentration, we applied BCA protocol. An equal amount of protein was electrophoresed using 10–12% resolving gel. Next, the transfer process was carried out using the PVDF membrane (EMD Millipore, Billerica, MA, USA). The membranes were blocked with 5% skimmed milk in 1x TBS-T for 1 hour. Subsequently, the membranes were incubated with primary and secondary antibodies. Finally, they were detected using the ECL reagent (Advansta, CA, USA) onto an X-ray film (Fuji Film, Tokyo, Japan).

### 2.11. Statistical Analyses

Each experiment was repeated at least three times in all cases. Data are presented as means ± SD (standard deviation). Statistical differences among groups were analyzed using GraphPad Prism 5. In all of the comparisons, a level of *p* < 0.05 was considered significant.

## 3. Results

### 3.1. Plasma Source and Plasma Properties

**Figure 1:**
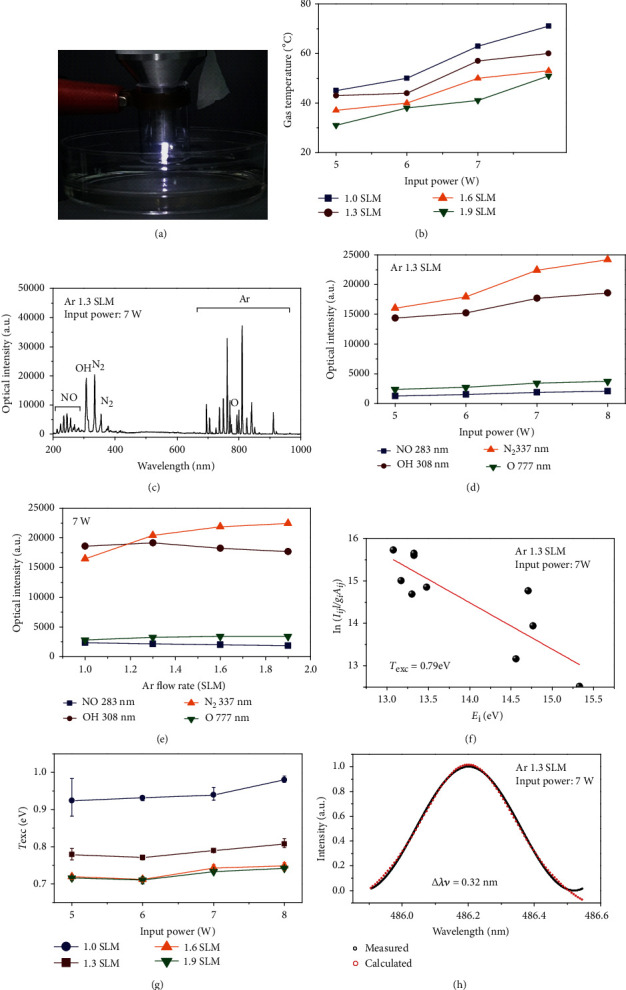
ME-APPJ device and plasma properties. (a) Photograph of microwave-excited atmospheric pressure argon plasma jet for plasma treatment on liquid. Diagnostics include optical emission spectroscopy. (b) Gas temperature vs. input power for different gas flow rates. (c) Optical emission spectrum from 200 to 1,000 nm observed in the ME-APPJ (input power of 7 W, gas flow rate of 1.3 SLM). Optical emission intensities of RONS-related lines NO (283 nm), OH (308 nm), O (777 nm), and N_2_ (337 nm) were compared at various input powers (d) and gas flow rates (e). (f) Boltzmann plots obtained from Ar lines for ME-APPJ (input power of 7 W, gas flow rate of 1.3 SLM). And (g) the changes of *T*_exc_ as a function of input power at different Ar gas flow rates. (h) Measured H*_𝛽_* line profile and the Voigt function fed to the normalized line profile points for ME-APPJ (input power of 7 W, gas flow rate of 1.3 SLM).

The ME-APPJ used for the plasma treatment on cell culture medium was represented in Figure 1(a). The gas temperature was measured 2 cm below the open end of ME-APPJ. As can be seen in Figure 1(b), the gas temperature was influenced by the input power and gas flow rate. For example, at the gas flow rate of 1.9 SLM, the gas temperature was increased from 31 up to 51°C as the input power was increased from 5 W to 8 W. And at the input power of 7 W, it went down to 41°C when the gas flow rate was 1.9 SLM. It indicates that the gas temperature can be controlled by the plasma operating parameters. When the plasma expands and collides with ambient air, it produces gaseous RONS such as OH, NO, O, and N_2_∗. To confirm produced radicals by plasma, a typical optical emission spectrum was measured from plasma and represented in Figure 1(c). ME-APPJ produces the NO*γ* bands (200–300 nm), the OH band (308 nm), the O line (777 nm), and N_2_ emission bands (300–440 nm) as well as excited Ar lines (500–1000 nm). In particular, the intensities of OH radicals were observed to be higher than those of other plasma sources reported previously [34].  Figure 1(d) shows the optical emission intensities at different input powers. It is observed that the emission intensities exhibit a monotonous increase with the input power, indicating that the ME-APPJ used in this study generates a stable plasma. On the other hand, gas flow dependence is quite complicated. As long as the flow is laminar, with the increase of the gas flow rate, the distance where the working gas is mixed with surrounding air also increases, which results in the higher inclusion of N_2_ and O_2_ in the plume [43]. Therefore, in Figure 1(e), with increasing flow rate, we observe a slight increase in the intensity of N_2_∗ and O, but slight decreases of OH and NO intensity. This seems to be caused by the decreases in electron temperature and gas temperature with an increasing flow rate. The RONS-related radicals generated by plasma can contribute to chemical reactions and result in the formation of short- and long-lived species in liquids or within cells. In these plasmas, since the electron-atom collisions and atom-atom collisions are the most important processes, the electron excitation temperature (*T*_exc_) increases with the electron temperature (*T*_e_) (although *T*_e_ is estimated to be a little higher than *T*_exc_) [44]. In Figures 1(f) and 1(g), we present the Boltzmann plots and the changes of *T*_exc_ as a function of input power at different Ar gas flow rates. *T*_exc_ was in the range of 0.71~0.98 eV. And the electron density is determined by the Stark broadening of the hydrogen Balmer *β* line (486.15 nm) as described in other works [35, 44]. The estimated electron density was approximately 5.36 × 10^14^ cm^−3^, as shown Figure 1(h).

### 3.2. Cytotoxic Effects of PAM on Various Cancer Cells and Normal Cells

**Figure 2:**
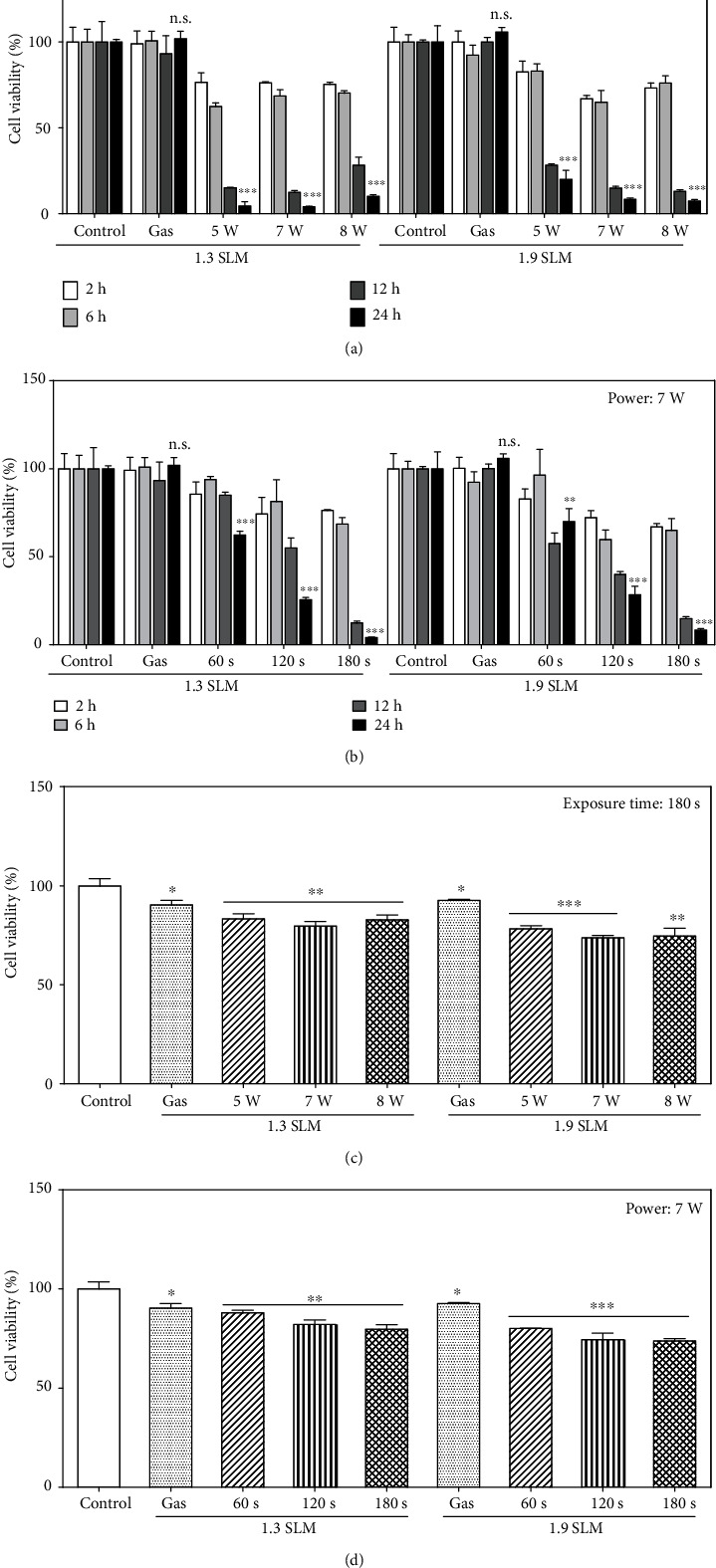
Cell viability of cancer cells (a, b) and normal cells (c, d). Cells were treated with the PAM produced at different conditions. The gas flow rates of plasma jet were 1.3 SLM and 1.9 SLM. Growth medium was exposed by plasma at 5, 7, and 8 W for 180 sec (a, c). In (b, d), plasma exposure time was 60, 120, and 180 sec at 7 W. Following 2, 6, 12, and 24 hours of PAM treatment, cancer cells were incubated with MTT solution and then measured by a microplate reader. Normal (Nuff) cells were treated with PAM for 24 hours. ^∗^*p* < 0.05, ^∗∗^*p* < 0.01, and ^∗∗∗^*p* < 0.001 vs. control. n.s. denotes *p* > 0.05 vs. controls.

RONS in PAM contribute to oxidative stress in the cell, which leads to cell death [45]. Thus, we investigated the cytotoxic effect of PAM on human lung (A549) cancer cells. As expected, PAM induced cell death of all the cancer cells that we tested in a dose-dependent manner (Figure 2). The effect of PAM produced under different conditions on the viability of A549 cells was evaluated at 2, 6, 12, and 24 hours post-PAM treatment. In Figures 2(a) and 2(b), cell viability was decreased with increasing PAM incubation time. However, the cell viability was not much affected by PAM up to 6 hours post PAM treatment, which indicates that PAM does not have an immediate effect on the viability of cells [46]. When the cell was treated by PAM for 24 hours, the cell viability decreased drastically but its dependence on input power and flow rate was not significant. Although it has been reported that PAM does not affect the viability of normal lung fibroblast cells [47, 48], we confirmed that PAM showed little cytotoxic effect on normal cells using additional normal cell line human foreskin fibroblast (Nuff). After the cells attached to the plate, PAM with the two different flow rate conditions was applied to Nuff cells for 24 hours. Figures 2(c) and 2(d) show the survival of Nuff cells. As a result, it showed much lower cytotoxicity than A549 cells. Taken together, it was confirmed that ME-APPJ-produced PAM has high toxicity to A549 cells than normal cells.

### 3.3. Measurement of Nitrite Concentration in PAM and PAM Incubated with A549 Cells

**Figure 3:**
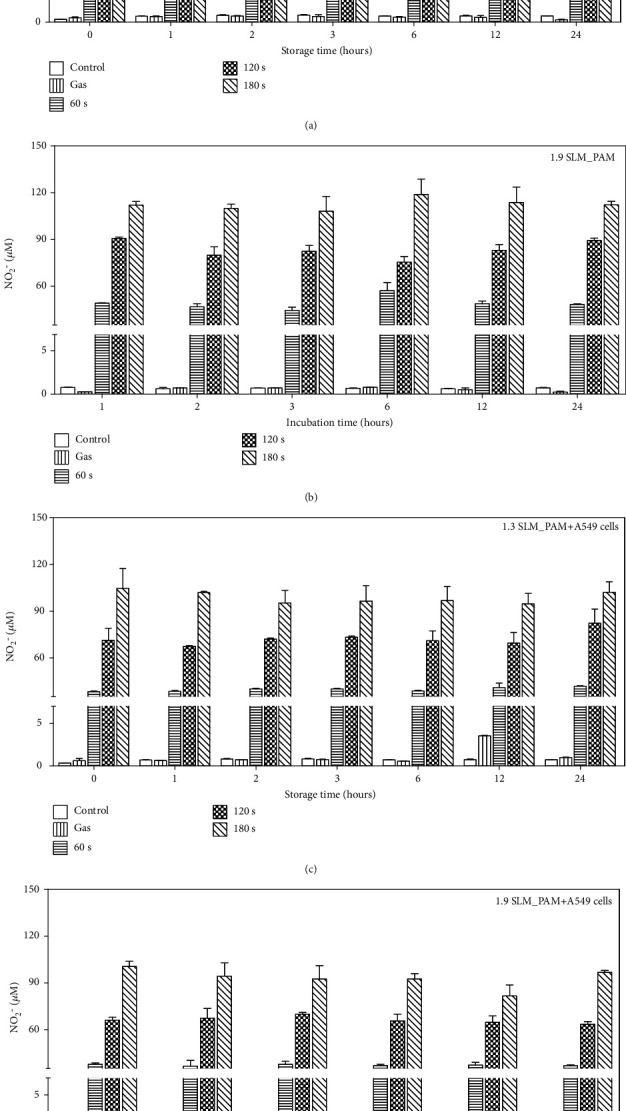
Measurement of NO_2_^−^ concentration in PAM. The PAM was produced at the conditions; the input power of 7 W, the gas flow rates 1.3 SLM and 1.9 SLM, and plasma exposure time 0–180 sec. NO_2_^–^ levels were measured immediately and 1, 2, 3, 6, 12, and 24 hours after plasma treatment in the stored PAM and the PAM incubated with A549 cells. Comparison of the NO_2_^–^ levels in the stored PAM (a, b) and in the PAM incubated with A549 cells (c, d) for different incubation times.

The concentration of NO_2_^–^ was measured in the stored PAM and the PAM incubated with A549 cells for different input power, plasma exposure times, and gas flow rate. The measurement of NO_2_^–^ concentration was performed immediately (0 hour) and 1, 2, 3, 6, 12, and 24 hours after plasma exposure. The NO_2_^–^ levels in the PAM increase with plasma exposure time, while it decreases slightly with the gas flow rate (Figures 3(a) and 3(b)). As shown in Figures 3(c) and 3(d), the NO_2_^–^ levels in the PAM including A549 cells were kept at a similar level compared to those in the PAM without cells, which indicates that the presence of cells does not influence the nitrite concentration. Also, it was observed that NO_2_^–^ levels in the PAM with and without cells remained not much changed during the storage or incubation period.

### 3.4. H_2_O_2_ Measurement of Plasma-Activated Medium

**Figure 4:**
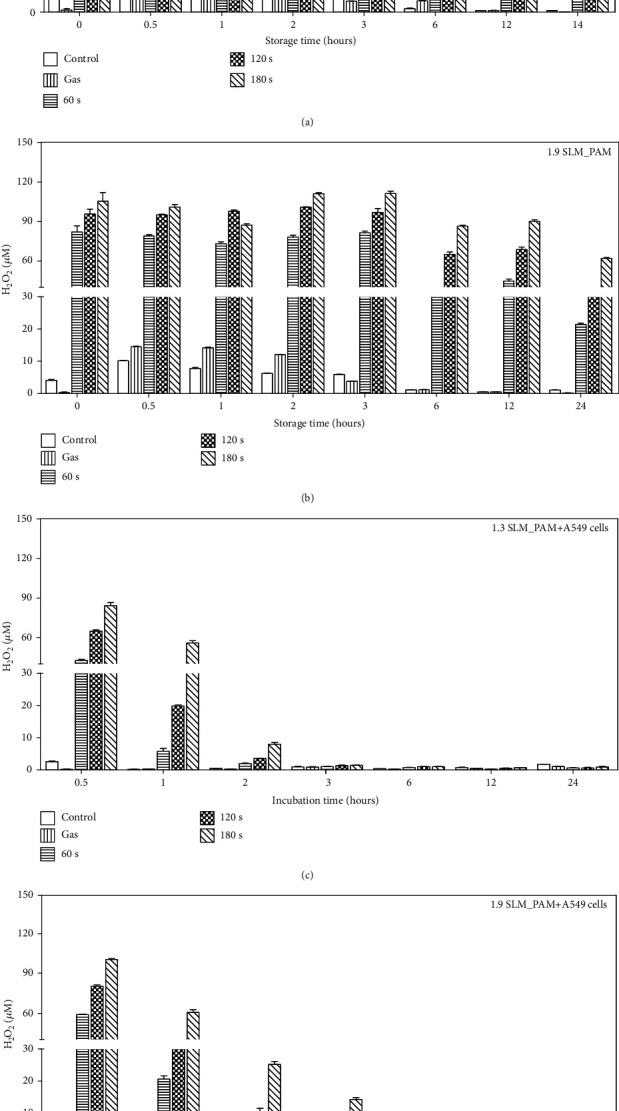
Measurement of H_2_O_2_ concentration in PAM. Amplex red hydrogen peroxide/peroxidase assay kit was used to measure H_2_O_2_ change in the PAM. The PAM was produced at the conditions; the gas flow rates 1.3 SLM and 1.9 SLM, the input power of 7 W, and the exposure times 60, 120, and 180 sec. H_2_O_2_ concentration was measured immediately and 0.5, 1, 2, 3, 6, 12, and 24 hours after plasma treatment on growth medium. Figures (a, b) show the H_2_O_2_ levels in the stored PAM. Figures (c, d) show the H_2_O_2_ concentration of treated PAM on A549 cells. After the cell treatment, the concentration of H_2_O_2_ in PAM was measured using Amplex red fluorescence (Ex/Em = 544/590 nm).

The concentration of H_2_O_2_ in the PAM was detected at 0, 0.5, 1, 2, 3, 6, 12, and 24 hours after plasma exposure for various operating conditions of plasma treatment. The H_2_O_2_ in the PAM is mainly provided by the plasma-generated H_2_O_2_. The gaseous H_2_O_2_ is produced via recombination of OH species or via HO_2_ (O_2_ + H⟶HO_2_; HO_2_ + HO_2_⟶H_2_O_2_ + O_2_).  Figure 4(a) shows the evolution of the measured fluorescence of H_2_O_2_ in the stored PAM and in the PAM incubated with the A549 cells produced at input power 7 W with different flow rates (1.3 and 1.9 SLM) and three plasma exposure times (60, 120, and 180 sec). As shown in Figure 4, the concentration of H_2_O_2_ in PAM is observed to increase with the plasma exposure time. These results suggest that there exists a strong correlation between cell viability and H_2_O_2_ in the PAM. Considering the NO_2_^–^ and H_2_O_2_ concentrations in the PAM applied to cancer cells shown in Figures 3 and 4, it can be stated that H_2_O_2_ mainly contributes to the death of cancer cells after the indirect cold plasma irradiation on the culture medium, as previously suggested [31, 32].

### 3.5. Intracellular ROS Measurement of A549 Cells and Nuff Cells Treated with PAM

**Figure 5:**
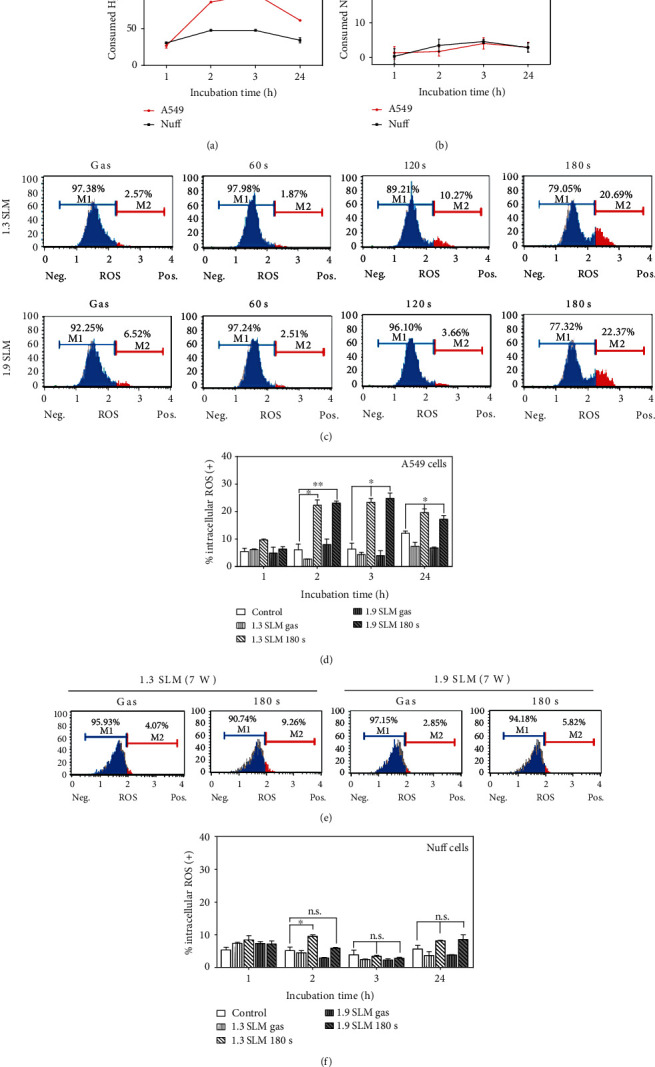
Detection of intracellular ROS in A549 and Nuff cells. (a) H_2_O_2_ concentration was measured 1, 2, 3, and 24 hours after incubation with PAM. Consumed NO_2_^–^ or H_2_O_2_ concentration (*μ*M) was calculated as concentration of storage PAM–concentration of incubated PAM with the cells. (b) NO_2_^–^ levels were measured 1, 2, 3, and 24 hours after plasma treatment in the PAM incubated with cells. (c) Histogram showed the intracellular ROS generation after PAM treatment on A549 cells for 2 hours. (d) Bar graphs showed the quantitative analyses of the intracellular ROS after PAM treatment for 1, 2, 3, and 24 hours. Intracellular ROS of Nuff cells treated with PAM for 2 hours (e) and bar graphs (f). The plasma exposure conditions were two flow rates (1.3 SLM and 1.9 SLM), three exposure times (60, 120, and 180 sec), and input power 7 W. ^∗^*p* < 0.05, ^∗∗^*p* < 0.01, and ^∗∗∗^*p* < 0.001 vs. control. n.s. denotes *p* > 0.05 vs. controls.

The concentration of H_2_O_2_ gradually decreases in A549-treated PAM due to self-degradation (Figure 5(a)), while no significant change of H_2_O_2_ or NO_2_^–^ concentration was observed in PAM treated to Nuff cells (Figure 5(b)). A drastic decrease of H_2_O_2_ concentration in the PAM applied to the cancer cells is attributed to the consumption of H_2_O_2_ in oxidizing cancer cells [23, 37]. The consumption is completed within 3 hours, and the concentration level remains thereafter. Damage to the plasma membrane by H_2_O_2_ not only increases its permeability to extracellular reactive species but also induces apoptosis [27, 28]. To measure the intracellular ROS level of A549 cells and Nuff cells by PAM, we used the Muse oxidative kit. Negative staining is referred to as M1 (ROS-), whereas the cells stained with the ROS-specific dye are referred to as M2 (ROS+). When PAM was applied to A549 cells for 2 hours (Figure 5(a)), intracellular ROS was increased in an exposure time-dependent manner. The positive ROS level was highest when culture medium was exposed by plasma at 7 W for 180 sec. Furthermore, the intracellular ROS level increased rapidly from 2 hours after incubation (Figure 5(b)). As a result, PAM can raise intracellular ROS levels in A549 cells from 2 hours, and ROS are maintained for up to 24 hours. On the other hand, when Nuff cells were treated with PAM, intracellular ROS generation was lower than A549 cells (Figures 5(c) and 5(d)). Through these results, it was confirmed that H_2_O_2_ generated from PAM had more influence on intracellular ROS generation in A549 cells than in normal cells.

### 3.6. PAM Promotes Cell Death of A549 Cells via Induction of Apoptosis and Cell Cycle Change

**Figure 6:**
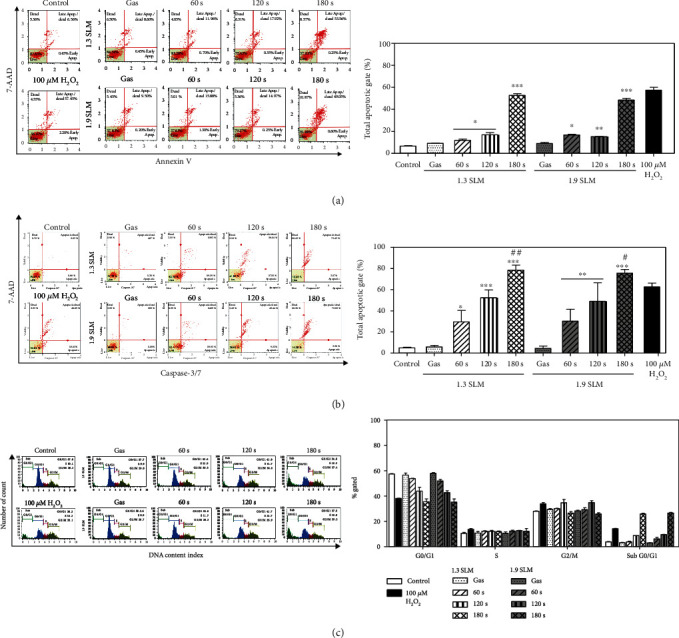
Induction of apoptosis in A549 cells by PAM. The percentage of the apoptotic cells was determined using the Muse Cell Analyzer. Medium irradiated with plasma at 7 W was applied to A549 cells for 24 hours. The distribution of apoptosis in A549 cells was measured using Annexin V/Dead Cell Assay (a) and caspase-3/7 detection reagent (b). (c) Histograms show the effect of PAM on cell cycle profile. ^∗^*p* < 0.05, ^∗∗^*p* < 0.01, and ^∗∗∗^*p* < 0.001 vs. control. ^#^*p* < 0.05 and ^##^*p* < 0.01 vs. 100 *μ*M H_2_O_2_.

To evaluate the apoptosis-inducing capability of PAM in A549 cells, the cells were treated with PAM for 24 hours. Thereafter, apoptosis was measured by the Muse Cell Analyzer. As shown in Figure 6(a), PAM-induced apoptosis in A549 cells depends on the plasma exposure time. In the PAM produced at the condition of 1.3 SLM, 7 W, and 180 sec, the ratio of A549 cells undergoing total apoptotic cells was increased from 11.91 ± 2.4% to 52.76 ± 0.06% after incubation for 24 hours. To determine pathways of apoptosis induced in the A549 cells, the expression level of caspase-3/7 and the change of cell cycle were examined by flow cytometry. In Figure 6(b), the results showed a similar tendency observed in the Annexin V staining. The total percentage of apoptotic cells with activated caspase-3/7 was 29.61 ± 1.59%, 52.39 ± 0.04%, and 78.54 ± 0.01% for the plasma exposure time of 60, 120, and 180 sec (at 1.3 SLM and 7 W), respectively, and 30.24 ± 1.73%, 49.01 ± 1.18%, and 75.7 ± 0.01% for the plasma exposure time of 60, 120, and 180 sec (at 1.9 SLM and 7 W), respectively. As shown in Figure 6(c), A549 cells incubated with PAM showed a reduction of cells in G0/G1, whereas the percentage of cells in the sub-G0/G1 phases was increased in a dose-dependent manner. These results indicate that apoptosis induced by PAM is related to cell cycle arrest.

### 3.7. PAM Regulates Phosphorylation of JNK and p53 and Activation of Bax

**Figure 7:**
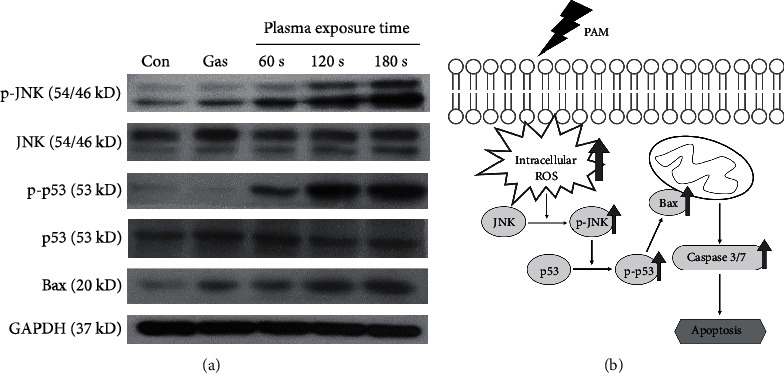
Activation of JNK/p53/Bax signaling in A549 cells by PAM. The A549 cells were treated for 0.5 h by PAM produced at the 1.9 SLM and 7-watt conditions. The protein expression change of JNK, p53, and Bax in PAM-treated A549 cells was measured by western blot analysis (a). (b) PAM reduces cell viability and induces apoptosis in A549 cells through the JNK/p53/Bax and caspase signaling pathway.

In order to investigate the underlying mechanism of the effects of PAM on A549 cells, western blot analysis was used to measure the expression levels of JNK, p53, and Bax. First, JNK is known to increase the phosphorylation level by ROS [49]. Observing that the intracellular ROS level of A549 cells was increased by PAM treatment in Figure 4, we checked the phosphorylation expression change of JNK. As shown in Figure 7(a), p-JNK expression was induced dependently on plasma exposure time. Furthermore, we investigated whether p53, which is known as a subsignal of JNK, is involved in the effects of PAM on apoptosis [50]. The results indicated that the phosphorylation of p53 was increased. In addition, phosphorylated p53 can induce caspase signaling by increasing the amount of Bax [51]. In the result of Figure 6(b), we already showed that PAM can activate caspase-3/7 in A549 cells. To confirm this result, we evaluated the expression level of Bax. PAM significantly induced Bax protein expression levels in A549 cells. Taken together, PAM can induce apoptosis by regulating the expression change of JNK/p53/Bax signaling.

## 4. Discussion

It was observed that ME-APPJ treatment induced a significant increase of RONS in media. The generated RONS in the PAM were measured by a colorimetric assay and compared with gaseous RONS in the plasma measured by optical emission spectroscopy [52, 53]. Plasma generation of reactive species appears to be governed by the total energy (input power × plasma exposure time). The gas temperature tended to increase as the input power increased and to decrease as the gas flow rate increased. Previous studies reported that four long-lived reactive species (H_2_O_2_, NO_2_^–^, NO_3_^–^, and O_3_) were dominant in PAM [54]. The present study investigated the effects of NO_2_^–^ and H_2_O_2_ in the PAM with A549 human lung cancer cells. The results revealed that in the case of the PAM without cancer cells, the concentrations of both species were decreased with the storage time. However, reduction of NO_2_^–^ was not affected by incubation with A549 cells. On the other hand, the concentration of H_2_O_2_ in the PAM with A549 cells was observed to decrease rapidly according to the incubation time. Furthermore, we observed that intracellular ROS was increased rapidly from 2 hours after A549 cells were treated with the PAM. These results suggest that hydrogen peroxide in the PAM influenced the increase of the intracellular ROS level in A549 cells.

Additionally, viability of A549 cells was weakly affected by input power and gas flow of the PAM but strongly affected by plasma exposure time. The indirect treatment by the ME-APPJ-produced PAM results in considerable cell death, indicating that PAM has a comparable killing effect comparable to that by the direct treatment of APPJ. We confirmed that cell death of A549 cells was induced via apoptosis using the Annexin V staining assay. Further, through some additional experiments, we have discussed the pathways of apoptosis. Firstly, the cell cycle analysis in the present study indicates a decrease of G0/G1 and an increase of sub-G0/G1, confirming that apoptosis may result from cell cycle arrest. Vedakumari et al. showed that BV-ERB-FN-treated A549 cells exhibited efficient cell cycle arrest with a decrease in the G0/G1 phase and an increase in the sub-G0/G1 phase resulting in apoptosis of cancer cells [55]. Secondly, our study showed that the expression level of caspase-3/7 was increased after the PAM treatment in the A549 cells. Caspase plays a central role in the process of apoptosis. Among them, caspase-3/7, which acts as an effector caspase, promotes apoptosis by receiving signals from higher caspase [56]. It has also been reported that a caspase-3-dependent apoptosis pathway is induced by cell cycle arrest [57]. And it was found that caspase-3/7 was activated at a higher rate by the PAM containing certain concentration of H_2_O_2_ than when only H_2_O_2_ at corresponding concentrations was added to the A549 cell culture. Finally, this study demonstrated that PAM induce apoptosis in human lung cancer cells through regulation of the JNK/p53/Bax signaling pathway. JNK/p53/Bax protein is a major factor known to induce apoptosis. Using western blot analysis, we revealed that elevated intracellular ROS induced phosphorylation of JNK, and p-JNK also increased phosphorylation of p53. As a result, Bax protein expression was increased. Eventually, this signaling pathway leads to apoptosis following caspase-3/7 activation (Figure 7(b)).

In anticancer studies using the PAM, it is reported that the PAM can induce the apoptosis signaling in cancer cells. In human cervical cancer cells, CAP induced oxidative stress on the mitochondria, increasing the transmembrane potential and promoting the release of apoptosis factor, which is regulated by the Bcl-2 family, which eventually activates caspase cascade [58]. Arndt et al. reported that a CAP device utilizing surface microdischarge technology showed anticancer effect in melanoma cells. CAP treatment induced DNA damage and regulated cell cycle, leading to increased apoptosis. It also induced proapoptosis events such as cytochrome C release, p53 and Rad 17 phosphorylation, and caspase-3 activation [16]. Moreover, PAM has been reported to inhibit metastasis of ovarian cancer cells. It was found that it reduced the expression of MMP-9 and prevented the activation of the MAPK pathway by inhibiting phosphorylation of JNK 1/2 and p38 MAPK [59]. Thus, although there are many reports on the PAM-induced apoptosis pathway, there is a lack of basic evidence.

In our study, it was demonstrated that hydrogen peroxide in PAM effectively increases intracellular ROS of A549 cells and induces cell cycle arrest, caspase-3/7 activation, and JNK/p53/Bax signaling regulation, ultimately leading to apoptosis. Even if the intracellular ROS increased rapidly from 2 hours, cell death takes some time. The reason is that being ROS the upper mediator, it takes time for the lower mediators to receive signals and change their expression. In fact, ROS are toxic to certain cells when present in higher concentration. Thus, PAM may exert additive effect on anticancer activity by adding more ROS into the cells, when the endogenous level of ROS itself is not enough to induce the cell death of the cancers. As shown in Figure 5, the cell viability decreased after 6 hours because cells need enough time to accumulate factors that cause apoptosis. In normal cells, on the other hand, PAM-induced intracellular ROS levels were low, resulting in lower cell cytotoxicity. The selectivity may be due to the difference in consumption of H_2_O_2_ into cancer cells and normal cells. The results are very suggestive of a correlation between the production of gaseous RONS in the plasmas and aqueous RONS in the PAM (and its cellular effect). The result implies that the PAM produced by ME-APPJ gives a controllable production of RONS and the standardized plasma source employed in this work can contribute to the validation and reproducibility of cancer therapy by PAM.

## 5. Conclusions

We have shown that PAM produced by ME-APPJ had a significant cytotoxic effect on A549 cells but had little effect on normal cell viability. In particular, hydrogen peroxide has been shown to play an important role in inducing anticancer effects. In this study, we present the rationale for the fundamental effect of PAM on cancer cells. Cell death of A549 cells was mainly via apoptosis, which occurred due to a higher level of intracellular ROS and was accompanied by cell cycle arrest and caspase-3/7 activation. Altogether, these results suggest that CAP therapies based on ME-APPJ-produced PAM may be a good consideration for human lung cancer treatment.

## Data Availability

Most of the data can be found in the manuscript, and further data used to support the results of this study may also be requested from the corresponding authors.

## References

[1] Shome D., von Woedtke T., Riedel K., Masur K. (2020). The HIPPO transducer YAP and its targets CTGF and Cyr61 drive a paracrine signalling in cold atmospheric plasma-mediated wound healing. *Oxidative Medicine and Cellular Longevity*.

[2] Tornin J., Mateu-Sanz M., Rodríguez A., Labay C., Rodríguez R., Canal C. (2019). Pyruvate plays a main role in the antitumoral selectivity of cold atmospheric plasma in osteosarcoma. *Scientific Reports*.

[3] Lee J. W., Han S. J., Kang H. Y., Wi S. S., Jung M. H., Kim K. S. (2019). On-off switching of cell cycle and melanogenesis regulation of melanocytes by non-thermal atmospheric pressure plasma-activated medium. *Scientific Reports*.

[4] Sagwal S. K., Pasqual-Melo G., Bodnar Y., Gandhirajan R. K., Bekeschus S. (2018). Combination of chemotherapy and physical plasma elicits melanoma cell death via upregulation of SLC22A16. *Cell Death & Disease*.

[5] Kaushik N. K., Ghimire B., Li Y. (2018). Biological and medical applications of plasma-activated media, water and solutions. *Biological Chemistry*.

[6] Cordaro L., De Masi G., Fassina A. (2019). The role of thermal effects in plasma medical applications: biological and calorimetric analysis. *Applied Sciences*.

[7] Isbary G., Heinlin J., Shimizu T. (2012). Successful and safe use of 2 min cold atmospheric argon plasma in chronic wounds: results of a randomized controlled trial. *The British Journal of Dermatology*.

[8] Shaw P., Kumar N., Kwak H. S. (2018). Bacterial inactivation by plasma treated water enhanced by reactive nitrogen species. *Scientific Reports*.

[9] Lou B. S., Lai C. H., Chu T. P. (2019). Parameters affecting the antimicrobial properties of cold atmospheric plasma jet. *Journal of Clinical Medicine*.

[10] Akhavan B., Michl T. D., Giles C. (2018). Plasma activated coatings with dual action against fungi and bacteria. *Applied Materials Today*.

[11] Arndt S., Unger P., Wacker E. (2013). Cold atmospheric plasma (CAP) changes gene expression of key molecules of the wound healing machinery and improves wound healing in vitro and in vivo. *PLoS One*.

[12] Arndt S., Unger P., Berneburg M., Bosserhoff A. K., Karrer S. (2018). Cold atmospheric plasma (CAP) activates angiogenesis-related molecules in skin keratinocytes, fibroblasts and endothelial cells and improves wound angiogenesis in an autocrine and paracrine mode. *Journal of Dermatological Science*.

[13] Hartwig S., Doll C., Voss J. O., Hertel M., Preissner S., Raguse J. D. (2017). Treatment of wound healing disorders of radial forearm free flap donor sites using cold atmospheric plasma: a proof of concept. *Journal of Oral and Maxillofacial Surgery*.

[14] Ratovitski E. A., Cheng X., Yan D. (2014). Anti-cancer therapies of 21st century: novel approach to treat human cancers using cold atmospheric plasma. *Plasma Processes and Polymers*.

[15] Li Y., Pan J., Ye G. (2017). In vitro studies of the antimicrobial effect of non-thermal plasma-activated water as a novel mouthwash. *European Journal of Oral Sciences*.

[16] Arndt S., Wacker E., Li Y. F. (2013). Cold atmospheric plasma, a new strategy to induce senescence in melanoma cells. *Experimental Dermatology*.

[17] Azzariti A., Iacobazzi R. M., Di Fonte R. (2019). Plasma-activated medium triggers cell death and the presentation of immune activating danger signals in melanoma and pancreatic cancer cells. *Scientific Reports*.

[18] Nguyen N., Park H., Hwang S., Lee J.-S., Yang S. (2019). Anticancer efficacy of long-term stored plasma-activated medium. *Applied Sciences*.

[19] Judée F., Fongia C., Ducommun B., Yousfi M., Lobjois V., Merbahi N. (2016). Short and long time effects of low temperature plasma activated media on 3D multicellular tumor spheroids. *Scientific Reports*.

[20] Ishaq M., Kumar S., Varinli H. (2014). Atmospheric gas plasma-induced ROS production activates TNF-ASK1 pathway for the induction of melanoma cancer cell apoptosis. *Molecular Biology of the Cell*.

[21] Turrini E., Laurita R., Stancampiano A. (2017). Cold atmospheric plasma induces apoptosis and oxidative stress pathway regulation in T-lymphoblastoid leukemia cells. *Oxidative Medicine and Cellular Longevity*.

[22] Duchesne C., Frescaline N., Lataillade J. J., Rousseau A. (2018). Comparative study between direct and indirect treatment with cold atmospheric plasma on *in vitro* and *in vivo* models of wound healing. *Plasma Medicine*.

[23] Takahashi Y., Taki Y., Takeda K. (2018). Cytotoxicity of cancer HeLa cells sensitivity to normal MCF10A cells in cultivations with cell culture medium treated by microwave-excited atmospheric pressure plasmas. *Journal of Physics D: Applied Physics*.

[24] Joh H. M., Choi J. Y., Kim S. J., Chung T. H., Kang T.-H. (2015). Effect of additive oxygen gas on cellular response of lung cancer cells induced by atmospheric pressure helium plasma jet. *Scientific Reports*.

[25] Jo A., Joh H. M., Chung J. W., Chung T. H. (2020). Cell viability and measurement of reactive species in gas- and liquid-phase exposed by a microwave-excited atmospheric pressure argon plasma jet. *Current Applied Physics*.

[26] Keidar M. (2015). Plasma for cancer treatment. *Plasma Sources Science and Technology*.

[27] Schmidt A., Bekeschus S., Jarick K., Hasse S., von Woedtke T., Wende K. (2019). Cold physical plasma modulates p 53 and mitogen-activated protein kinase signaling in keratinocytes. *Oxidative Medicine and Cellular Longevity*.

[28] Hasse S., Seebauer C., Wende K. (2019). Cold argon plasma as adjuvant tumour therapy on progressive head and neck cancer: a preclinical study. *Applied Sciences*.

[29] Mohades S., Barekzi N., Razavi H., Maruthamuthu V., Laroussi M. (2016). Temporal evaluation of the anti-tumor efficiency of plasma-activated media. *Plasma Processes and Polymers*.

[30] Lu P., Boehm D., Bourke P., Cullen P. J. (2017). Achieving reactive species specificity within plasma-activated water through selective generation using air spark and glow discharges. *Plasma Processes and Polymers*.

[31] Furuta R., Kurake N., Ishikawa K. (2017). Intracellular responses to reactive oxygen and nitrogen species, and lipid peroxidation in apoptotic cells cultivated in plasma-activated medium. *Plasma Processes and Polymers*.

[32] Kurake N., Tanaka H., Ishikawa K. (2017). Effects of •OH and •NO radicals in the aqueous phase on H2O2and $\text{NO}_{2}^{-}$ generated in plasma-activated medium. *Journal of Physics D: Applied Physics*.

[33] Girard P. M., Arbabian A., Fleury M. (2016). Synergistic effect of H_2_O_2_ and NO_2_ in cell death induced by cold atmospheric He plasma. *Scientific Reports*.

[34] Kurake N., Tanaka H., Ishikawa K. (2016). Cell survival of glioblastoma grown in medium containing hydrogen peroxide and/or nitrite, or in plasma-activated medium. *Archives of Biochemistry and Biophysics*.

[35] Choi J., Eom I. S., Kim S. J. (2017). Characterization of a microwave-excited atmospheric-pressure argon plasma jet using two-parallel-wires transmission line resonator. *Physics of Plasmas*.

[36] Yan D., Cui H., Zhu W. (2017). The strong cell-based hydrogen peroxide generation triggered by cold atmospheric plasma. *Scientific Reports*.

[37] McKay K., Iza F., Kong M. G. (2010). Excitation frequency effects on atmospheric-pressure helium RF microplasmas: plasma density, electron energy and plasma impedance. *European Physical Journal D: Atomic, Molecular, Optical and Plasma Physics*.

[38] Choi J., Iza F., Do H. J., Lee J. K., Cho M. H. (2009). Microwave-excited atmospheric-pressure microplasmas based on a coaxial transmission line resonator. *Plasma Sources Science and Technology*.

[39] Chen Z., Liu X., Zou C. (2017). Donut shape plasma jet plumes generated by microwave pulses even without air mole fractions. *Journal of Applied Physics*.

[40] Jiang J., Tan Z., Shan C. (2016). A new study on the penetration of reactive species in their mass transfer processes in water by increasing the electron energy in plasmas. *Physics of Plasmas*.

[41] Miotk R., Hrycak B., Jasinski M., Mizeraczyk J. (2012). Spectroscopic study of atmospheric pressure 915 MHz microwave plasma at high argon flow rate. *Journal of Physics Conference Series*.

[42] Kim H., Jo A., Baek S. (2017). Synergistically enhanced selective intracellular uptake of anticancer drug carrier comprising folic acid-conjugated hydrogels containing magnetite nanoparticles. *Scientific Reports*.

[43] Lu X., Naidis G. V., Laroussi M., Ostrikov K. (2014). Guided ionization waves: theory and experiments. *Physics Reports*.

[44] Zhu X.-M., Chen W. C., Pu Y. K. (2008). Gas temperature, electron density and electron temperature measurement in a microwave excited microplasma. *Journal of Physics D: Applied Physics*.

[45] Šantak V., Zaplotnik R., Tarle Z., Milošević S. (2015). Optical emission spectroscopy of an atmospheric pressure plasma jet during tooth bleaching gel treatment. *Applied Spectroscopy*.

[46] Van Loenhout J., Flieswasser T., Boullosa L. F. (2019). Cold atmospheric plasma-treated PBS eliminates immunosuppressive pancreatic stellate cells and induces immunogenic cell death of pancreatic cancer cells. *Cancers*.

[47] Nguyen N. H., Park H. J., Yang S. S., Choi K. S., Lee J. S. (2016). Anti-cancer efficacy of nonthermal plasma dissolved in a liquid, liquid plasma in heterogeneous cancer cells. *Scientific Reports*.

[48] Torii K., Yamada S., Nakamura K. (2015). Effectiveness of plasma treatment on gastric cancer cells. *Gastric Cancer*.

[49] Kim M. O., Moon D. O., Jung J. M., Lee W. S., Choi Y. H., Kim G. Y. (2011). *Agaricus blazei* extract induces apoptosis through ROS-dependent JNK activation involving the mitochondrial pathway and suppression of constitutive NF-*κ*B in THP-1 cells. *Evidence-based Complementary and Alternative Medicine*.

[50] Jo H., Ahn H. J., Lee J. H., Min C. K. (2015). Roles of JNK and P 53 in taxol-induced apoptotic signaling in SKOV3 human ovarian cancer cells. *Elyns Journal of Cancer Research*.

[51] Amaral J. D., Xavier J. M., Steer C. J., Rodrigues C. M. (2010). The role of p 53 in apoptosis. *Discovery Medicine*.

[52] Mohades S., Laroussi M., Sears J., Barekzi N., Razavi H. (2015). Evaluation of the effects of a plasma activated medium on cancer cells. *Physics of Plasmas*.

[53] Saadati F., Mahdikia H., Abbaszadeh H. A., Abdollahifar M. A., Khoramgah M. S., Shokri B. (2018). Comparison of direct and indirect cold atmospheric-pressure plasma methods in the B_16_F_10_ melanoma cancer cells treatment. *Scientific Reports*.

[54] Lu X., Naidis G. V., Laroussi M., Reuter S., Graves D. B., Ostrikov K. (2016). Reactive species in non-equilibrium atmospheric-pressure plasmas: generation, transport, and biological effects. *Physics Reports*.

[55] Vedakumari S. W., Senthil R., Sekar S., Babu C. S., Sastry T. P. (2019). Enhancing anti-cancer activity of erlotinib by antibody conjugated nanofibrin-in vitro studies on lung adenocarcinoma cell lines. *Materials Chemistry and Physics*.

[56] Chandler J. M., Cohen G. M., MacFarlane M. (1998). Different subcellular distribution of caspase-3 and caspase-7 following Fas-induced apoptosis in mouse liver. *The Journal of Biological Chemistry*.

[57] Han S., Shin H., Lee J. K. (2019). Secretome analysis of patient-derived GBM tumor spheres identifies midkine as a potent therapeutic target. *Experimental & Molecular Medicine*.

[58] Ahn H. J., Kim K. I., Kim G., Moon E., Yang S. S., Lee J. S. (2011). Atmospheric-pressure plasma jet induces apoptosis involving mitochondria via generation of free radicals. *PLoS One*.

[59] Nakamura K., Peng Y., Utsumi F. (2017). Novel intraperitoneal treatment with non-thermal plasma-activated medium inhibits metastatic potential of ovarian cancer cells. *Scientific Reports*.

